# Improving Nausea Through Breathing Interventions: A Trial of Written Instructions for Diaphragmatic Breathing Versus a Biofeedback Device

**DOI:** 10.1155/cjgh/2341938

**Published:** 2026-01-25

**Authors:** Subhankar Chakraborty

**Affiliations:** ^1^ Department of Gastroenterology, Hepatology and Nutrition, The Ohio State University, Columbus, Ohio, USA, osu.edu

**Keywords:** anxiety, biofeedback, breathing exercise, depression, diaphragmatic breathing, gastrointestinal, nausea

## Abstract

**Background:**

Nausea is a distressing symptom affecting ∼7% of the population. Pharmacologic options are limited and often ineffective for chronic nausea. Nonpharmacologic strategies, such as breathing exercises, have shown promise in reducing stress and GI symptoms, but their role in chronic nausea has not been studied. This study addresses this knowledge gap by comparing written diaphragmatic breathing (DB) instructions with a biofeedback device (CalmiGo) for their effect on nausea severity.

**Methods:**

In a prospective study, we investigated the effects of breathing exercises using either written instructions for DB or a biofeedback respiratory practice device (CalmiGo) on self‐reported severity of nausea. Participants were randomized to either intervention and asked to practice the exercises three times daily for 3 min each time for 6 Weeks. Nausea was evaluated at baseline and every week for 7 Weeks via online self‐reported surveys.

**Results:**

A total of 85 adults with nausea (*n* = 44, 51.7% severe nausea) were randomized to either DB (*n* = 36) or CalmiGo (*n* = 49). There was no difference between the two groups at baseline in demographic features, anxiety, depression, or nausea severity. Nausea improved at all‐time points in both groups with a medium to large effect size. However, after applying false discovery rate correction, the improvement remained significant for DB only at weeks one to three and borderline significant at weeks four to five and Week 7. There was no difference in response rates between the two groups. Age, body mass index, baseline anxiety, and whether one was diabetic were predictive of improvement in nausea after 4 Weeks.

**Conclusion:**

In a pilot study, we observed that brief breathing exercises improve nausea. Breathing exercises may be useful as a nonpharmacologic option in the management of nausea, although larger trials are needed to confirm these findings.

## 1. Background

Nausea is a distressing symptom affecting approximately 7% of the population. Pharmacologic options are limited and often ineffective for chronic nausea [1]. Nonpharmacologic strategies to alleviate nausea have been reported to be somewhat effective in acute nausea such as in the postoperative period and during pregnancy [2]. These include ginger, lavender oil, and wearable transcutaneous electrical neurostimulators [2–4]. Breathing exercises, specifically diaphragmatic breathing (DB) and slow deep breathing, have shown promise in reducing stress and GI symptoms in conditions like gastroesophageal reflux and rumination syndrome [5–7]. However, their role in chronic nausea has not been investigated. This study addresses this knowledge gap by comparing written DB instructions with a biofeedback device (CalmiGo) for their effect on nausea severity.

## 2. Methods

This was a prospective study conducted at a tertiary university in the Midwestern United States. Participants were enrolled from across the United States. The protocol was approved by the Institutional Review Board at our university (Study ID: 2022H0256). To be eligible, participants had to be at least 18 years of age, understand English, have moderate‐to‐severe symptoms of anxiety or depression, and have moderate‐to‐severe symptoms of gastroparesis (i.e., nausea, early satiety, postprandial fullness, or abdominal pain) [8]. Those who could not understand English were excluded. While to be eligible, they had to have any one of these GI symptoms; for this study, we focus on nausea. The sensation of nausea was rated on a scale ranging from 0 (none) to 4 (very severe) [9]. Anxiety and depression symptoms were assessed using the Hospital Anxiety and Depression Survey (HADS). Based on the HADS score, anxiety, and depression symptoms were categorized as absent (0–7), mild (8–10), moderate (11–14), or severe (15–21). To be eligible, participants had to have an HADS‐anxiety or HADS‐depression score of 11 or more [10].

After enrollment, participants completed a baseline survey about demographics, after which they were randomized to either the device arm (CalmiGo) or to the written instruction arm (received a PDF instruction on how to perform DB exercises). The randomization was computer generated and based on their age, sex, BMI, and diabetes history. Blinding was not feasible due to the nature of the intervention. Investigators were aware of group allocation. The CalmiGo group was encouraged to complete a 5‐min training phone call to supplement the written instructions on using the device that were provided. Both groups were asked to practice breathing exercises thrice a day for 3 min at a time for 6 Weeks. CalmiGo is a small handheld device that guides users to extend their exhalation using multisensory cues (light, vibration, and sound). It was used as an alternative format for breathing training in this study. At the end of each week, participants completed electronic surveys regarding the severity of nausea. The final survey was sent to participants 1 Week after finishing the exercises (i.e., in Week 7) [8].

We did not perform a formal power calculation to determine the sample size for this study. Given the exploratory nature of the analysis and the limited available data, the sample size was based on feasibility rather than statistical power which is a limitation of our study design as the study may be underpowered to detect small but potentially meaningful effects.

All data were entered into REDCap. De‐identified data were first downloaded as an Excel file and then analyzed using SPSS Version 29.0 (IBM Corporation, USA) and visualizations created with help of the Data Analyzer App (https://data-analysis-master.replit.app/). A modified intention‐to‐treat analysis was conducted that included all those who completed the baseline survey. The primary outcome was the weekly change in nausea severity from baseline to Week 4. Continuous data were expressed as mean with standard deviation. Categorical data were expressed as frequency and percentage. Continuous data were compared between groups using an independent *t*‐test (if there were 2 groups) or one‐way ANOVA (if > 2 groups). Paired *t*‐tests were used to compare changes in nausea severity at each follow‐up week relative to baseline. To account for multiple comparisons, the false discovery rate (FDR) correction using the Benjamini–Hochberg procedure was applied. Given the exploratory nature of our study, we felt that this approach helps balance the risk of false positives with the need to detect potentially meaningful effects. Effect size was calculated using Cohen’s d. The effect size was categorized as small (0 to −0.20), medium (−0.21 to −0.50), and large (−0.51 to −1.0). Categorical data were compared using the chi‐square test. Univariate logistic regression was employed to examine if demographics or baseline symptoms had any association with who responded to breathing exercises. A responder was defined as anyone who had at least a one‐point decrease in nausea. All others were considered nonresponders. All results were expressed with 95% confidence intervals where applicable. *p* values were provided for all statistical tests. A *p* value of less than 0.05 was considered statistically significant.

This study was not registered on ClinicalTrials.gov. At the time of study initiation, the project was designed as a small‐scale, exploratory pilot intended to assess feasibility and generate preliminary data rather than to test a predefined hypothesis in a confirmatory manner.

## 3. Results

**Figure 1:**
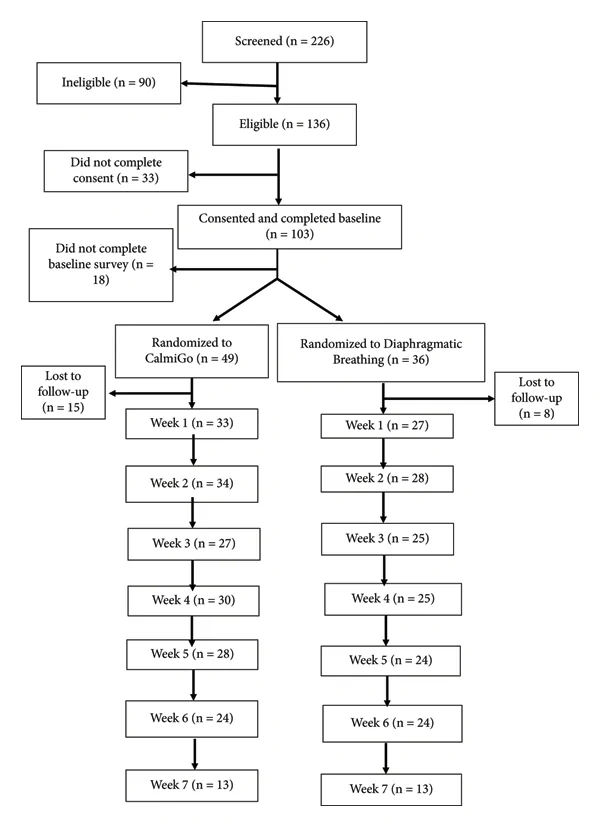
CONSORT flow diagram of participant progress through the study. A total of 226 participants were screened, of whom 90 were ineligible. Among 136 eligible participants, 33 did not provide consent and 18 did not complete the baseline survey, leaving 85 participants who were randomized (CalmiGo: *n* = 49; Diaphragmatic Breathing: *n* = 36). Weekly retention numbers are displayed for each arm. Loss to follow‐up occurred in 15 participants in the CalmiGo arm and eight in the diaphragmatic breathing arm. Reasons for loss to follow‐up, where available, included voluntary withdrawal and inability to re‐establish contact.

A total of 226 individuals were screened, of whom 90 were found ineligible. Among the 136 eligible participants, 33 did not complete consent. Of the 103 who consented, 18 did not complete the baseline survey, leaving 85 participants who were randomized to either the CalmiGo group (*n* = 49) or the DB group (*n* = 36). A CONSORT flow diagram of participant progress through the study is shown in Figure 1.

### 3.1. Baseline Characteristics

At baseline, 44 participants reported severe or very severe, 20 moderate, and 17 mild nausea. There was no difference in demographic characteristics like age (*p* = 0.41), race (*p* = 0.67), sex (*p* = 0.17), or body mass index (BMI, *p* = 0.60) between the DB and CalmiGo groups. There was also no difference in HADS‐anxiety (*p* = 0.72) or depression scores (*p* = 0.24) or in the proportion of diabetes (*p* = 0.16). The two groups were similar in the baseline severity of nausea (*p* = 0.26) and in the proportion of patients with severe or very severe nausea, as shown in Table 1.

**Table 1:** **Baseline Characteristics of Participants by Intervention Group** Comparison between CalmiGo device users and those receiving diaphragmatic breathing instructions.

	CalmiGo® (*N* = 49) Mean (SD)	Diaphragmatic breathing (*N* = 36) Mean (SD)	*p* value
Age	41.08 (13.89)	43.47 (12.14)	0.411
White race, *n* (%)	47 (95.9%)	32 (88.9%)	0.675
Female sex, *n* (%)	39 (79.6%)	32 (88.9%)	0.169
BMI	28.37 (9.07)	27.35 (8.05)	0.601
HADS‐anxiety	14.15 (2.92)	13.89 (3.56)	0.717
HADS‐depression	9.89 (4.55)	11.03 (4.03)	0.240
Nondiabetic, *n* (%)	40 (81.6%)	34 (94.4%)	0.158
Baseline nausea severity	2.29 (1.24)	2.56 (0.97)	0.264
Severe or very severe nausea, *n* (%)	24 (48.9%)	20 (55.5%)	0.772

*Note:* Values are presented as mean with standard deviation (SD) for continuous variables and number with percentage in parenthesis for categorical variables. *p* values reflect group comparisons using *t*‐tests or chi‐square tests.

Abbreviations: BMI = body mass index, HADS = Hospital Anxiety and Depression Scale.

**Figure 2:**
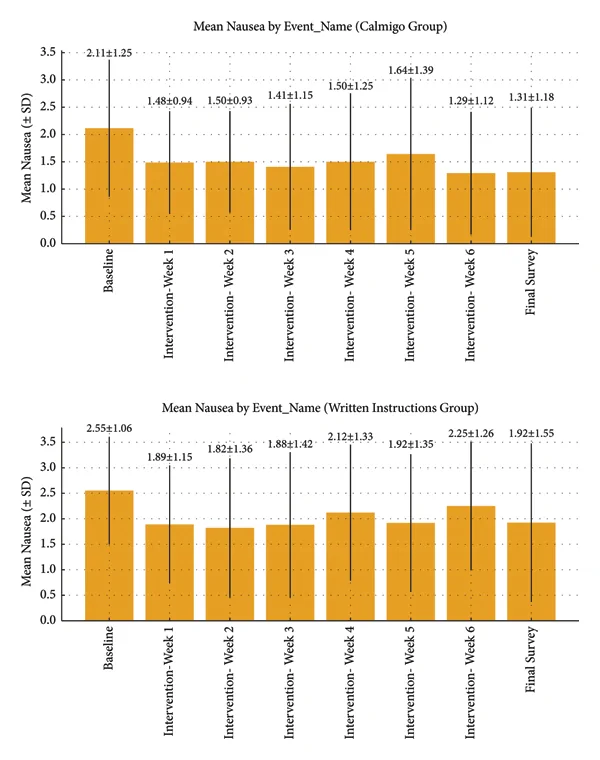
Mean severity of nausea at baseline and during intervention. The figure presents the average nausea scores (± standard deviation) across different time points for two intervention groups. Top panel: Participants using the CalmiGo device. Bottom panel: Participants following diaphragmatic breathing instructions. The *x*‐axis represents the study timeline, including baseline, weekly intervention periods, and the final survey. The *y*‐axis shows the mean nausea scores. Each bar indicates the mean nausea severity for that time point, with error bars representing standard deviation.

In the CalmiGo group, reductions in nausea severity were observed each week compared to baseline (Figure 2), with several comparisons reaching statistical significance based on raw *p* values. However, after applying the FDR correction to account for multiple comparisons, these differences were no longer statistically significant (FDR‐adjusted *p* values > 0.05). The effect size was mostly medium (Cohen’s *d* ranging from −0.40 to −0.52), with a large effect size seen at Week 6 (*d* = −0.57) and Week 7 (*d* = −0.74).

In the DB group, nausea severity decreased consistently across all time points (Figure 2). After applying the FDR correction, statistically significant improvements were observed during Weeks 1 through 3 (FDR‐adjusted *p* values < 0.01). Effect sizes were medium to large (Cohen’s *d* = −0.52 to −0.91).

There was no difference in the degree of change in nausea between the CalmiGo and DB groups at matching time points (Table 2).

**Table 2:** Change in nausea severity from baseline at clinically meaningful timepoints.

Time	*N*	Change from baseline	Effect size	Raw *p* value	FDR‐adjusted *p* value	*p* value CalmiGo® versus DB
CalmiGo®						
Δ Week 1	33	−0.576	−0.401	0.028	0.155	0.604
Δ Week 2	34	−0.588	−0.520	0.005	0.132	0.259
Δ Week 3	27	−0.519	−0.436	0.032	0.155	0.787
Δ Week 4	30	−0.500	−0.408	0.033	0.155	0.732
Δ Week 5	28	−0.393	−0.271	0.164	0.581	0.657
Δ Week 6	24	−0.708	−0.575	0.010	0.138	0.220
Δ Week 7	13	−0.846	−0.740	0.020	0.155	0.712
Diaphragmatic breathing (DB)						
Δ Week 1	27	−0.778	−0.799	< 0.0001	0.004	
Δ Week 2	28	−0.821	−0.908	< 0.0001	0.001	
Δ Week 3	25	−0.760	−0.751	0.001	0.009	
Δ Week 4	25	−0.520	−0.517	0.016	0.085	
Δ Week 5	24	−0.708	−0.518	0.018	0.085	
Δ Week 6	24	−0.333	−0.408	0.057	0.178	
Δ Week 7	13	−0.692	−0.731	0.022	0.087	

*Note:* This table summarizes the change in nausea severity from baseline at weeks one to seven for participants using either the CalmiGo device or diaphragmatic breathing (DB) techniques. N: number of participants at each timepoint. Change from baseline: mean reduction in nausea severity score. Effect size: Cohen’s d representing the magnitude of change. Raw *p* value: unadjusted significance level for within group change. FDR‐adjusted *p* value: false discovery rate correction for multiple comparisons. *p* value (CalmiGo vs. DB): between group comparison at each timepoint. Influence of baseline characteristics on improvement in nausea.

*CalmiGo:* Week‐4 responders had a significantly greater BMI (*p* = 0.0075) than nonresponders. They were also older than nonresponders, but this difference was not significant (*p* = 0.069). The proportion of males was higher in nonresponders (5 of 14 vs. an expected frequency of 2 out of 14, *p* = 0.033). There was no difference in baseline severity of nausea, HADS‐Anxiety, or HADS‐depression scores (Table 3). Race (White) and history of diabetes were also similar between groups (*p* = 0.94,  0.41 respectively, data not shown).

**Table 3:** Baseline characteristics of responders versus nonresponders to CalmiGo after 4 weeks.

	Responders (*n* = 16)	Nonresponders (*n* = 14)	*p* value	Effect size
Age	47.25 ± 12.60	37.57 ± 15.49	0.069	0.715
BMI	28.72 ± 7.17	22.22 ± 4.71	0.0075	1.092
Baseline nausea severity	2.38 ± 0.96	1.57 ± 1.45	0.081	0.687
HADS‐anxiety	13.62 ± 2.89	14.29 ± 2.78	0.530	−0.240
HADS‐depression	10.00 ± 4.80	10.14 ± 4.42	0.933	−0.032

*Note:* Values are presented as mean ± standard deviation. Responders were defined as participants who showed improvement in nausea severity by at least one point from baseline after 4 weeks of CalmiGo use. *p* values reflect group comparisons using *t*‐tests. Effect size is reported as Cohen’s d.

*DB:* No significant differences were observed between Week‐4 responders and nonresponders in age, BMI, baseline nausea severity, HADS‐anxiety, or HADS‐depression (Table 4). The proportions of White race, female sex, and diabetes history were likewise similar (*p* = 0.29, 0.15, and 1.00, respectively, data not shown).

**Table 4:** Baseline characteristics of Responders versus nonresponders to diaphragmatic breathing after 4 Weeks.

	Improved (*n* = 10)	Unchanged or worse (*n* = 15)	*p* value	Effect size
Age	43.2 ± 14.07	42.2 ± 9.99	0.83	0.089
BMI	28.02 ± 10.85	24.99 ± 6.88	0.40	0.366
Baseline nausea severity	2.60 ± 1.08	2.67 ± 1.11	0.88	−0.063
HADS‐anxiety	12.5 ± 4.30	14.8 ± 2.92	0.12	−0.681
HADS‐depression	10.8 ± 2.97	10.67 ± 4.10	0.93	0.037

*Note:* Values are presented as mean ± standard deviation. Responders were defined as participants who showed improvement in nausea severity by at least one point from baseline after 4 Weeks of diaphragmatic breathing. *p* values reflect group comparisons using *t*‐tests. Effect size is reported as Cohen’s d.

### 3.2. Baseline Participant Characteristics Associated With Improvement in Nausea Following Breathing Exercises

Among participants who completed at least 4 Weeks of intervention (across both the CalmiGo and DB groups), several baseline characteristics were significantly associated with improvement in nausea. Specifically, older age (OR = 1.04, *p*  <  0.0001), higher BMI (OR = 1.09, *p*  <  0.0001), and lower HADS‐anxiety scores (OR = 0.87, *p*  <  0.0001) were linked to a greater likelihood of improvement. Conversely, the presence of diabetes was associated with reduced odds of improvement (OR = 0.46, *p* = 0.0065). Other factors—including race, sex, baseline nausea severity, and intervention type (CalmiGo vs. written instructions for DB)—were not associated with improvement in nausea (see Table 5). Due to the limited sample size, multivariate regression was not performed, which is acknowledged as a limitation of this analysis.

**Table 5:** Univariate predictors of nausea improvement after 4 Weeks of breathing intervention.

	Odds ratio	95% C.I.	*p* value
Age	1.035	1.023–1.048	< 0.0001
Race (White)	0.710	0.412–1.226	0.22
Sex (male)	0.588	0.125–2.76	0.50
BMI	1.097	1.076–1.119	< 0.0001
Diabetes (yes)	0.461	0.264–0.805	0.0065
Baseline nausea severity	1.26	0.80–1.97	0.321
HADS‐anxiety	0.866	0.834–0.899	< 0.0001
HADS‐depression	0.994	0.140–7.055	0.995
Group (diaphragmatic breathing)	0.660	0.297–1.471	0.309

*Note:* Odds ratios reflect the likelihood of nausea improvement after 4 Weeks of intervention. Values are derived from univariate logistic regression models.

Abbreviations: BMI = body mass index, CI = confidence interval, DB = diaphragmatic breathing, HADS = Hospital Anxiety and Depression Scale.

## 4. Discussion

This exploratory study demonstrated that both written DB instructions and device‐guided breathing using CalmiGo were associated with reductions in nausea severity. The interventions were brief, feasible, and produced medium to large effect sizes, suggesting potential clinical benefit. Notably, there was no significant difference in effectiveness between the two approaches, underscoring the practical equivalence of a commercial device and a low‐cost, instruction‐based method.

Given the small sample size and exploratory nature of our analysis, we applied the FDR correction, after which the improvement in nausea was significant only for the DB group between Weeks 1–3 (*p* < 0.05) and borderline significant (0.1 < *p* < 0.05) between four and five and at Week 7. The FDR adjustment is designed to reduce the likelihood of false positives when multiple comparisons are made. However, in studies with small sample sizes, such as ours, the FDR correction can also increase the risk of false negatives—masking potentially meaningful effects. The fact that we observed a consistent direction (i.e., reduction in nausea severity from baseline at all‐time points) of change and a medium to large effect size suggests that the lack of significance may reflect limited statistical power rather than the absence of a true effect.

Logistic regression analysis revealed that individual characteristics influenced response to the intervention. Participants who were older, had higher BMI, had lower baseline anxiety scores, and had no history of diabetes were more likely to experience improvement in nausea. These findings suggest that personalized factors may play a role in the effectiveness of breathing‐based therapies and warrant further investigation.

While prior studies have demonstrated the benefit of breathing exercises in chemotherapy‐induced and postoperative nausea, evidence in chronic unexplained nausea and disorders of gut‐brain interaction (DGBIs) remains limited [2, 6, 11, 12]. Our study is one of the first to address this gap and supports further investigation into breathwork as a nonpharmacologic option for nausea management.

Despite the encouraging effect on nausea, our study has several limitations. The sample size was relatively small, and attrition rates were high, which limits the generalizability of the findings and reduces statistical power. Additionally, no formal power calculation was performed, which further constrains the interpretation of the results. The study relied exclusively on self‐reported outcomes without incorporating objective physiological measures, which may introduce bias or variability in symptom reporting. The absence of long‐term follow‐up prevents assessment of whether the observed benefits were sustained over time. Finally, there is potential bias introduced by the fact that participants in the CalmiGo group received device training and support, which was not available to those in the written‐instruction group.

A key practically helpful observation was that improvements in nausea were comparable between the device and written instruction groups. This suggests that patients may achieve benefit from DB practice without requiring a proprietary device, though some individuals may prefer or adhere better with a device‐based option.

## 5. Conclusion

In summary, our study provides preliminary evidence that both paced‐breathing devices and written DB instructions may reduce nausea symptoms and that response to breathing exercises is moderated by patient characteristics such as age, BMI, anxiety, and diabetes status. Given the small sample size, high dropout, and exploratory nature of the analyses, these results should be interpreted with caution and confirmed in larger, adequately powered trials with longer follow‐up and objective outcome measures.

## Disclosure

The funder had no role in the design, conduct, analysis, or manuscript preparation.

## Conflicts of Interest

The author declares no conflicts of interest.

## Funding

CalmiGo provided the devices used in the study.

## Data Availability

The data that support the findings of this study are available on request from the corresponding author. The data are not publicly available due to privacy or ethical restrictions.
